# Experimentally induced diabetes causes glial activation, glutamate toxicity and cellular damage leading to changes in motor function

**DOI:** 10.3389/fncel.2014.00355

**Published:** 2014-10-31

**Authors:** Aarti Nagayach, Nisha Patro, Ishan Patro

**Affiliations:** ^1^School of Studies in Neuroscience, Jiwaji UniversityGwalior, India; ^2^School of Studies in Zoology, Jiwaji UniversityGwalior, India

**Keywords:** STZ-induced diabetes, astroglia, microglia, cerebellum, behavior, glutamate transporter

## Abstract

Behavioral impairments are the most empirical consequence of diabetes mellitus documented in both humans and animal models, but the underlying causes are still poorly understood. As the cerebellum plays a major role in coordination and execution of the motor functions, we investigated the possible involvement of glial activation, cellular degeneration and glutamate transportation in the cerebellum of rats, rendered diabetic by a single injection of streptozotocin (STZ; 45 mg/kg body weight; intraperitoneally). Motor function alterations were studied using Rotarod test (motor coordination) and grip strength (muscle activity) at 2nd, 4th, 6th, 8th, 10th, and 12th week post-diabetic confirmation. Scenario of glial (astroglia and microglia) activation, cell death and glutamate transportation was gaged using immunohistochemistry, histological study and image analysis. Cellular degeneration was clearly demarcated in the diabetic cerebellum. Glial cells were showing sequential and marked activation following diabetes in terms of both morphology and cell number. Bergmann glial cells were hypertrophied and distorted. Active caspase-3 positive apoptotic cells were profoundly present in all three cerebellar layers. Reduced co-labeling of GLT-1 and GFAP revealed the altered glutamate transportation in cerebellum following diabetes. These results, exclusively derived from histology, immunohistochemistry and cellular quantification, provide first insight over the associative reciprocity between the glial activation, cellular degeneration and reduced glutamate transportation, which presumably lead to the behavioral alterations following STZ-induced diabetes.

## Introduction

Diabetes mellitus is a metabolic disorder well known for its austere impact on central nervous system. Cognitive and behavioral dysfunctions are being the most apparent CNS impairments reported in both humans (Petrofsky et al., 2005; Kodl and Seaquist, 2008) and animal models of diabetes (Biessels et al., 1994; Coleman et al., 2004; Guven et al., 2009). It is held that altered metabolism of lipids, proteins and carbohydrates following diabetes leads to oxidative stress and cell death in the brain, causing a state of dysfunctions in cognition and behavior. Patients with diabetes and hyperglycemia present poor motor coordination and reduced motor activity (Daneman, 2001; Cox et al., 2005; Petrofsky et al., 2005).

Cerebellum is involved in the control and execution of several aspects of motor functions, including coordination (Horne and Butler, 1995), muscle tone (Manto, 2010) and locomotion (Morton and Bastian, 2004). The cerebellar circuitry involves a distinctive single layer of Purkinje cells (PC) as the sole output neurons that receive input directly or indirectly from the climbing fibers and parallel mossy fibers. The precise input/output system of inhibitory and excitatory synaptic information enables the Purkinje cells to predominantly control and coordinate the cerebellar motor functions. Bergmann glia (radial form of astroglia), elaborately wrap the dendritic spines and excitatory synapses of Purkinje neurons and actively take part in the processing and maintenance of excitatory synaptic information via glutamate transporters (Rothstein et al., 1994; Bellamy, 2006). Compelling evidence indicated that astroglial cells possess high affinity glutamate transporters (GLT-1/GLAST) and are notably involved in maintaining the extracellular glutamate concentration at sub-excitotoxic levels via uptaking the excess of glutamate and thus prevent possible neuronal cell death due to glutamate toxicity (Rothstein et al., 1996; Anderson and Swanson, 2000). Furthermore, impairment in glutamate transporter GLT-1/GLAST were related to the neurodegenerative disorders like epilepsy, hypoxia/ ischemia, amyotrophic lateral sclerosis, Alzheimer's disease (AD), Parkinson disease (PD) and schizophrenia (Sims and Robinson, 1999; Danbolt, 2001).

Glia (astroglia and microglia) have a wide range of functions in CNS ranging from the regulation of innate immunity, scavenging dead cell debris, scaffolding and protecting neurons to the regulation and maintenance of synaptic transmission and putative integration of information with neurons. Following any brain insult or immune breaching, glial cells get activated and exhibit morphological transformations from resting to activated, increase in their cell population and secrete an array of inflammatory molecules (Patro et al., 2005; Luo and Chen, 2012; Boche et al., 2013). Glial activation based on the progression of insult and severity of stimulus, imposes both beneficial (Morgan et al., 2004; Luo and Chen, 2012) and detrimental effects (Heneka et al., 2010) upon the brain cells.

So far, only a couple of studies have elucidated the alterations in cellular mechanisms of cerebellum following diabetes (Hernández-Fonseca et al., 2009; Antony et al., 2010). However, to best of our knowledge, consequences were rarely correlated with the behavioral alterations that too with limitations. The potential effects of diabetes on the cerebellum are still poorly understood. Therefore, present study was aimed to investigate the influence and scenario of glial activation, cellular degeneration and glutamate transportation in cerebellum and associated behavioral patterns of the experimentally induced diabetic rats.

## Materials and methods

### Animals

All experiments were carried out using 3 month old male Wistar rats weighing 200–220 gm (School of Studies in Neuroscience, Jiwaji University, Gwalior), as their susceptibility to STZ-induced diabetes is previously well reported (McNeill, 1999). The care and maintenance of animals was carried out as per the guidelines of CPCSEA and all the experimental procedures were pre-approved by Institutional Animal Ethics Committee, Jiwaji University, Gwalior (India). The rats were housed in polypropylene cages having clean dust free husk with *ad libitum* access to food and water. Animals were maintained in controlled environment having temperature of 25 ± 2°C and 50–65% humidity with a fixed 12:12 h light dark cycle.

### Induction of diabetes

Type 1 diabetes was induced by a single intraperitoneal injection of 45 mg/kg body weight of Streptozotocin (STZ; Sigma) prepared in 0.1 M citrate buffer (pH = 4; Stevens et al., 2007; Lebed et al., 2008; Nagayach et al., 2014) to the overnight fasted rats. Control rats were injected with vehicle alone. Diabetes was confirmed at 72 h post STZ injection, by testing the blood glucose level (non-fasting) through tail snipping method using AccuChek Sensor Comfort (Roche Diagnostics, Berlin, Germany) and the animals having blood glucose level of 250 mg/dl or above were considered as diabetic. Animal's blood glucose level (non-fasting) and body weight were checked once a week upto 12 weeks to ensure the diabetic stature. Diabetic rats were randomly divided into following groups, i.e., 2nd, 4th, 6th, 8th, 10th,and 12th week post-diabetic confirmation. Subsequently animal's food and water consumption were also measured daily to further confirm the diabetic symptoms like polyphagia and polydipsia.

### Behavioral test

All the animals (*n* = 6/group) were subjected to behavioral test. Behavioral assessment for motor coordination and neuromuscular strength was achieved by rotarod test and grip strength meter respectively. The animals were subjected to the behavioral test on the aforementioned days of diabetic duration.

### Rotarod test

Rotarod test was performed as per previously described method (Kumar et al., 2013) with the help of Rotamex 5 (Columbus Instruments, USA). Animals were acclimatized for three consecutive days at start speed of 2 rpm and a maximum speed of 8 rpm for 100 s of duration. After 24 h of acclimatization, final reading was taken with acceleration time of 2 rpm (start speed) to 40 rpm (final speed) for 420 s as total time duration. An animal fall was detected by infrared photo-cells automatically with the help of software (Rotamex 5, Columbus Instruments, USA). Once the photocells lose the detection of the animal, the falling latency of that animal was recorded by the software attached with the apparatus. Falling latency is directly proportional to the riding time. Test animals were given 3 trials each with a resting time interval of 15 min each between the successive trials. Final trials were performed four times for each animal in every group, and the values were computed for single mean value for every animal. The experimental room conditions (light and temperature) and timing were consistent in all the trials.

### Grip strength

Grip strength meter (Columbus Instruments, USA) was used to detect the motor/ muscular function of the diabetic animal. The apparatus is consisted of a force gage digital display (sensor range: 0–5 kg) connected with specially designed forelimb grasping pull bar assemblies (76 × 50 mm) made up of steel wire. The values of grip strength were recorded automatically via RS-232 interface connected to the computer and a software Grip strength version 1.19. The test animal was placed carefully over the metallic grid and allowed to hold the grid through its forelimbs. Proper care was taken for applying gentle uniform force while animal was pulled back by holding the tail until its grip gets released. The maximum strength value animal takes to release the grid was displayed on the screen and was saved for further data analysis. Six trials were performed successively followed by 2 min resting interval, for each animal in every group and the values were computed for single mean value for every animal. The experimental room conditions (light and temperature) and timing were consistent in all the trials.

### Tissue collection and processing

Animals from both control and diabetic groups were sacrificed at the aforementioned time points, i.e., 2nd, 4th, 6th, 8th, 10th and 12th week. Under ether anesthesia perfusion was performed as per previously described methods (Patro et al., 2010b; Nagayach et al., 2014). Animals were perfused via transcardiac puncture, with 2% paraformaldehyde prepared in 0.01 M phosphate buffer (PB) after flushing with phosphate-buffered saline (PBS; 0.01 M; pH 7.4). After perfusion-fixation the brain was dissected out carefully, weighed and post-fixed in the same fixative overnight at 4°C. The tissues were then cryoprotected in Phosphate buffered-Sucrose gradients, i.e., 10, 20, and 30% at 4°C until tissue settled at the bottom. After processing the sagittal sections of cerebellum were cut serially using Microm HM 525 (Thermo Scientific) cryostat, at a thickness of 14 μm. The sections were collected on chrom alum-gelatin coated slides and stored at −20°C till they were used for immunohistochemical studies.

### Immunohistochemistry

Immunostaining was performed as per previously described methods (Patro et al., 2009; Nagayach et al., 2014). Briefly, cryocut sections were sequentially treated with 1% Triton X-100 and 1% hydrogen peroxide in washing buffer for 30 min each at room temperature followed by three washings (5 min each) with buffer. Non-specific proteins were blocked with 1% normal serum (dissolved in washing buffer) of same species as secondary antibody, for 90 min in a humid chamber. Following blocking, the sections were incubated with primary antibodies, GFAP (1:2000, Rabbit polyclonal, Dako, Denmark), Iba-1 (1:1500, Rabbit polyclonal, Wako Japan), Active caspase-3 (1:1000, Rabbit polyclonal, R&D Systems), at the specified titre overnight at 4°C. Next day, the sections were then incubated with appropriate secondary antibody anti-rabbit biotin-labeled (1:100, Sigma) for 90 min at room temperature. After three buffer washings (5 min each), sections were further incubated with streptavidin-biotin-peroxidase complex (1:200, Amersham) for 90 min at room temperature. Subsequent to buffer wash the color was developed with chromogen solution containing 3, 3′-diaminobenzidine tetrahydrochloride (0.025%) and hydrogen peroxide (0.006%) dissolved in the buffer for 20 min at room temperature. Nickel enhancement was done for caspase-3 labeling. Later on trailed by distilled water wash, slides were dried at 37°C, dehydrated in 100% alcohol, cleared in xylene, and mounted with DPX for further microscopic evaluations.

### Co-immunolabeling

The co-immunolabeling was done as per previously described method (Patro et al., 2010b). Followed by permeablization with 0.5% Triton X-100 and non-specific protein blocking with 5% normal serum (dissolved in washing buffer), sections were incubated with cocktail of primary antibodies GFAP (1: 2000; Rabbit polyclonal, Dako, Denmark) and GLT-1 (1: 1000; Guinea pig polyclonal, Chemicon) for overnight at 4°C to label the astroglia and glutamate transporters respectively. Next day, the sections were washed with PBS buffer and incubated with the cocktail of flurochrome conjugated secondary antibody Cy3 (1:300; anti-guinea pig, Vector) and Fluorescein isothiocynate (FITC; 1:400; anti-rabbit, Abcam) for the visualization of GLT-1 and GFAP respectively. Afterwards, sections were washed with PBS buffer for four times (10 min each) to remove the excess of secondary antibody and mounted with antifade VECTASHIELD® HardSet™ mounting medium with DAPI (Vector Laboratories, Burlingame, CA).

To ensure comparable immunostaining, sections were processed together at the same time in the same conditions. Omission of primary antibodies served as negative control.

Assessment of morphologic alterations and quantification of the expression level of various proteins was achieved with quantitative immunohistochemical analysis simultaneously on the similar platform in the brain section. Furthermore, phenotypic assessment also provided concrete information about the specific cyto- and/or histo-morphological change, which is mostly required in explaining the glial activation and allied subcellular and vascular deformations. Undoubtedly Western blot analysis would have been more useful but could not be performed due to limitations of animal use.

### Histological study

For histological study a separate set of animals from each group were perfused and processed for paraffin sectioning as per previously described method (Kumar et al., 2013). Briefly, after perfusion-fixation, tissues were thoroughly washed with water and dehydrated with graded series of ethyl alcohol. Then, the tissues were cleared in toluene and infiltrated in Paraplast (Sigma, m. p. 56–58°C) for proper impregnation of wax. Tissue blocks were made in paraffin and serial sagittal sections of cerebellum were cut at a thickness of 6 μm using Leica RM2135 microtome.

To observe the histological changes, tissue sections were stained with 0.1% cresyl violet acetate (Sigma certified stain, C-5042), prepared in acetate buffer (pH 3.5) and routinely used Delafield's Haematoxylin and eosin stain.

### Image analysis

Bright field images were captured by using Leica DM 6000 microscope equipped with Leica DFC 310 FX digital camera and Leica Application Suite (LAS V4.2) software in order to have magnified images showing fine structural details for more demarcated morphological analysis. Fluorescent co-immunolabeled sections were visualized using a Leica DM 6000 microscope with digital camera (Leica DFC 310 FX) operating with the Leica Application Suite Advanced Fluorescence (LAS AF, Leica) and images were grabbed using I3 and N2.1 filters for FITC and Cy3 respectively and overlaid with the overlay module of Leica Application Suite Advanced Fluorescence.

### Cell quantification and analysis

Cell quantification was done on the sections stained through standard HRP-conjugated immunohistochemistry method. The cell population was estimated in all the ten folias of the cerebellum. The cerebellar regions for quantification were delineated as three frames per folia in the two sections per animal in all the groups. Tissue sections corresponding to the region of interest were included in the quantification ensuring the equivalence in the procedure. In order to count equal areas (mm^2^) in every subject, a 23,299.6 μm^2^ frame comprising exclusively the anatomical areas of interest was applied to ensure that counts were representative of the analyzed areas. Photomicrographs of the regions were photographed with Leica Laborlux microscope fitted with digital camera (Leica DFC 420 DC). Cell counting was performed on the digital pictures with Leica Qwin software (Version 3.1) application interactive measure (IM) and presented as the total number of cells/mm^2^. The area fraction was measured with NIH ImageJ software (http://rsb.info.nih.gov/ij/download.html).

### Statistical analysis

Results were statistically analyzed with SigmaStat software 3.5 version and were reported as mean ± s.e.m. In experiments, unpaired *t*-test was used to assess the significance between the two groups, and with more than two groups, assessment was done by One-Way ANOVA followed by the Tukey's *post-hoc* test. The threshold for statistical significance was set at *p* ≤0.05. In figures ^*^p ≤ 0.01, ^**^p ≤ 0.001.

## Results

### STZ induction resulted in characteristic diabetes

**Figure 1:**
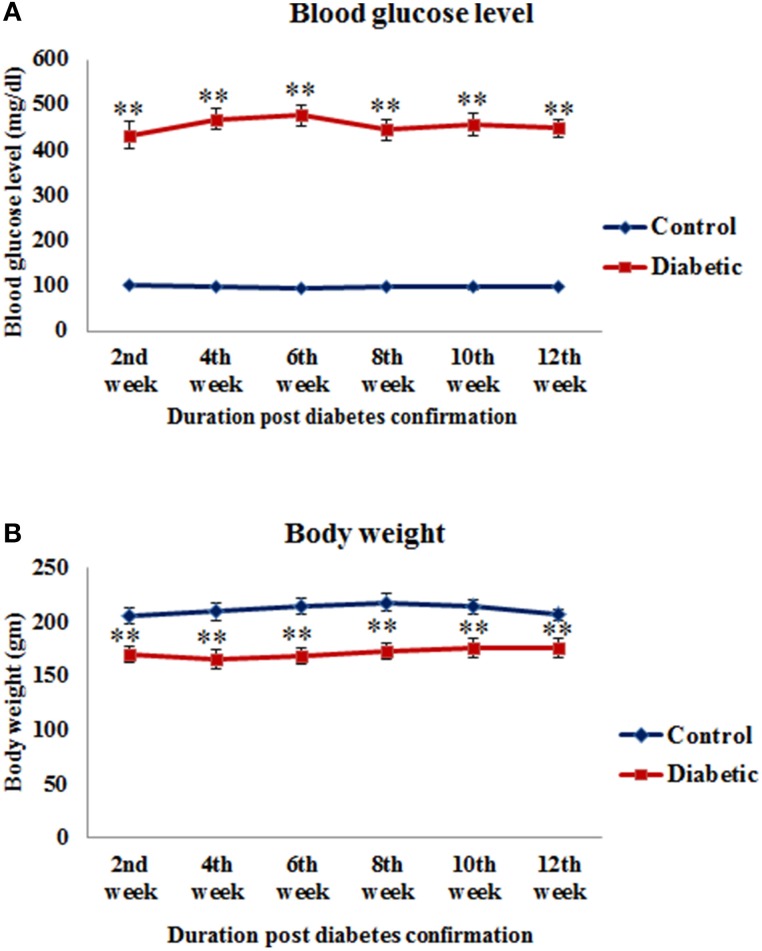
**Blood glucose level (A) and body weight (B) of control and STZ-induced diabetic animals**. Diabetic animals were showing significantly reduced body weight and increased blood glucose level in comparison to controls. Values are presented as mean ± s.e.m. (*n* = 6.8). ^**^p ≤ 0.001 for comparison of diabetic group with the respective controls.

All the STZ-induced animals exhibited characteristic signs of diabetes as their blood glucose level was significantly high in 2nd week [*t*_(14)_ = −12.165, *p* ≤ 0.001], 4th week [*t*_(14)_ = −20.013, *p* ≤ 0.001], 6th week [*t*_(14)_ = −18.467, *p* ≤ 0.001], 8th week [*t*_(14)_ = −11.689, *p* ≤ 0.001], 10th week [*t*_(10)_ = −14.964, *p* ≤ 0.001] and 12th week [*t*_(10)_ = −16.339, *p* ≤ 0.001] time points of diabetes as compared to controls (Figure 1A). Similarly, the food consumption and water intake also increased in the diabetic animals presenting symptoms of polyphagia and polydipsia. Body weight was found to be significantly reduced in 2nd week [*t*_(14)_ = 5.692, *p* ≤ 0.001], 4th week [*t*_(14)_ = 5.439, *p* ≤ 0.001], 6th week [*t*_(14)_ = 5.959, *p* ≤ 0.001], 8th week [*t*_(14)_ = 5.963, *p* ≤ 0.001], 10th week [*t*_(10)_ = 3.762, *p* ≤ 0.001] and 12th week [*t*_(10)_ = 3.729, *p* ≤ 0.001] in diabetic animals upto the 12th weeks (Figure 1B).

### Behavioral assessment

#### Rotarod test

**Figure 2:**
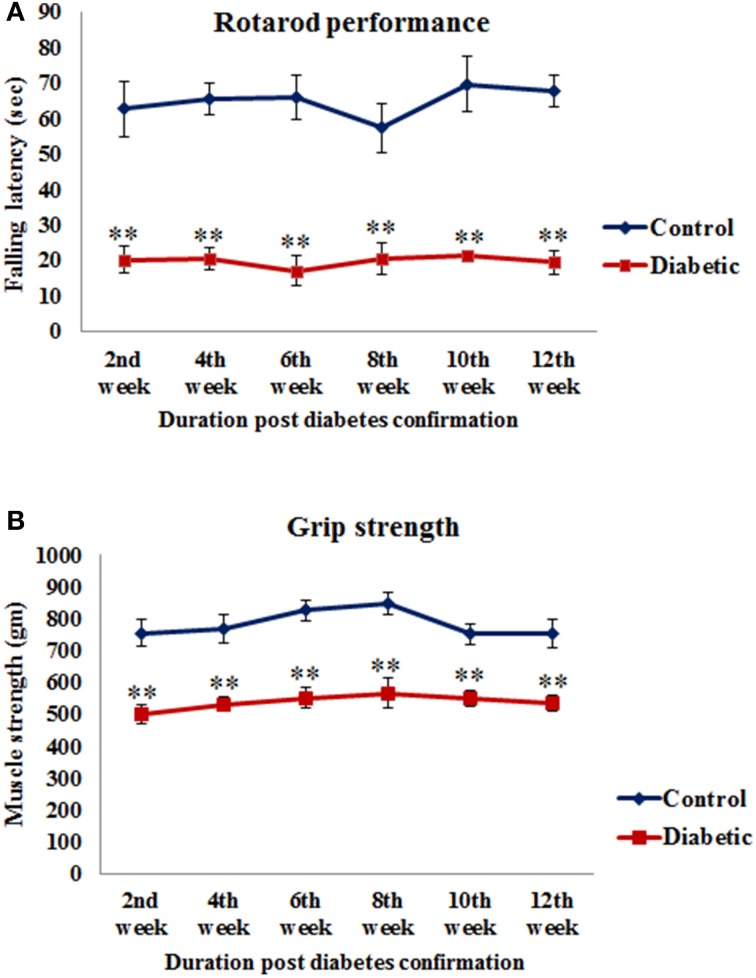
**Alteration in motor functions following STZ-induced diabetes**. **(A)** On rotarod performance diabetic animals presented significantly reduced motor coordination on accelerating rod in comparison to controls. **(B)** Grip strength assessment data showed significantly weak grip strength of diabetic animals in comparison to controls. Values are presented as mean ± s.e.m. (*n* = 6). ^**^p ≤ 0.001 for comparison of diabetic group with the respective controls.

Motor coordination was assessed as total length of time spent by the animals on the rotating rod. The falling latency of diabetic animals was significantly decreased in 2nd week [*t*_(10)_ = 4.865, *p* ≤ 0.001], 4th week [*t*_(10)_ = 8.22, *p* ≤ 0.001], 6th week [*t*_(10)_ = 6.531, *p* ≤ 0.001], 8th week [*t*_(10)_ = 4.526, *p* ≤ 0.001], 10th week [*t*_(10)_ = 6.085, *p* ≤ 0.001] and 12th week [*t*_(10)_ = 8.575, *p* ≤ 0.001] of diabetic time points as compared to the respective controls (Figure 2A).

#### Grip strength

A significant reduction was observed in the grip strength of all the diabetic animals and the values were significant at all the time points studied, i.e., 2nd week [*t*_(10)_ = 4.885, *p* ≤ 0.001], 4th week [*t*_(10)_ = 4.658, *p* ≤ 0.001], 6th week [*t*_(10)_ = 5.970, *p* ≤ 0.001], 8th week [*t*_(10)_ = 4.641, *p* ≤ 0.001], 10th week [*t*_(10)_ = 4.926, *p* ≤ 0.001] and 12th week [*t*_(10)_ = 4.339, *p* ≤ 0.001] as compared to the respective controls (Figure 2B) indicating a reduced muscle strength following STZ-induced diabetes.

#### Effect of diabetes on glial cells

**Figure 3:**
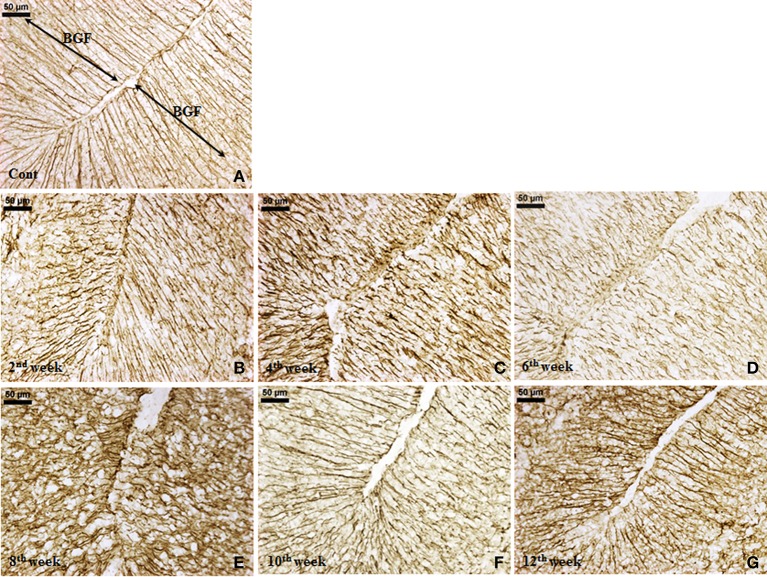
**Effect of diabetes on Bergmann glial fibers (BGF)**. GFAP immunolabeled Bergmann glial fibers (BGF) of cerebellum were presenting a disorganized and hypertrophied morphology **(B–G)** at all the diabetic time points in comparison to controls having thin, intact and erect morphology **(A)**. Scale bar = 50 μm.

**Figure 4:**
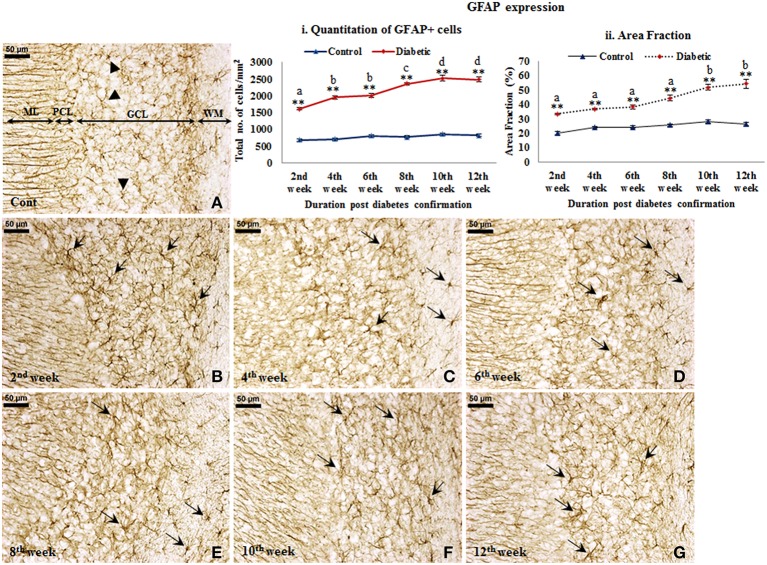
**Persistent astroglial activation following diabetes**. GFAP immunolabeled light microscopic images of both diabetic and control cerebellum depicting profound astroglial activation during diabetes. Astroglial activation was consistent in white matter and in all the three layers of the cerebellum upto the 12th week of diabetic duration bearing thick, dense, wavy and fragmented processes with a darkly stained large cell body (**B–G**; arrows). In controls astroglia were presenting resting morphology having thin, fine processes and lightly stained small cell body (**A**; arrowheads). ML, Molecular layer; PCL, Purkinje cell layer; GCL, Granule cell layer; WM, White matter. Scale bar = 50 μm. Quantitation and area fraction graphs were showing a significantly increased population (i) and volumetric fraction (ii) of GFAP + cells in the diabetic cerebellum upto the 12 weeks post-diabetic confirmation in comparison to controls. Same alphabets on bars indicate non-significant differences between groups at the given time points (*p* ≤ 0.001). Value represents mean ± s.e.m. of the 30 readings/ animal/ time point. ^**^p ≤ 0.001 for comparison of diabetic group with the respective controls.

***GFAP expression.*** An increased GFAP expression in the diabetic cerebellum upto 12th week of diabetes was immunohistochemically observed. In controls, the GFAP labeled Bergmann glial fibers were presenting intact, thin and erect morphology (Figure 3A) while in diabetic animals Bergmann glial fibers became hypertrophied, fragmented and disorganized at all the time points (Figures 3B–G). Similarly, the astroglia in the white matter and granule cell layer also presented activated morphology having thick, dense and fragmented processes with the darkly stained cell body as compared to resting astroglia with thin processes and lightly stained cell body in controls (Figure 4). Quantitation of GFAP positive cells showed a significant gradual increment (*p* ≤ 0.001) in astroglia population at all the diabetic points, i.e., 2nd week [*F*_(11,359)_ = 18.548, *p* ≤ 0.001], 4th week [*F*_(11,359)_ = 24.187, *p* ≤ 0.001], 6th week [*F*_(11,359)_ = 23.972, *p* ≤ 0.001], 8th week [*F*_(11,359)_ = 31.600, *p* ≤ 0.001], 10th week [*F*_(11,359)_ = 33.203, *p* ≤ 0.001] and 12th week [*F*_(11,359)_ = 33.246, *p* ≤ 0.001] as compared to the respective controls. A progressive escalation was also observed in the cerebellar astroglial population following diabetes (Figure 4i). Volumetric fraction (area fraction) assessment also showed a significant increment in the percentage of GFAP expression at all the diabetic points, i.e., 2nd week [*F*_(11,359)_ = 9.292, *p* ≤ 0.001], 4th week [*F*_(11,359)_ = 8.904, *p* ≤ 0.001], 6th week [*F*_(11,359)_ = 9.714, *p* ≤ 0.001], 8th week [*F*_(11,359)_ = 13.042, *p* ≤ 0.001], 10th week [*F*_(11,359)_ = 16.788, *p* ≤ 0.001] and 12th week [*F*_(11,359)_ = 19.489, *p* ≤ 0.001] as compared to the respective controls (Figure 4ii).

**Figure 5:**
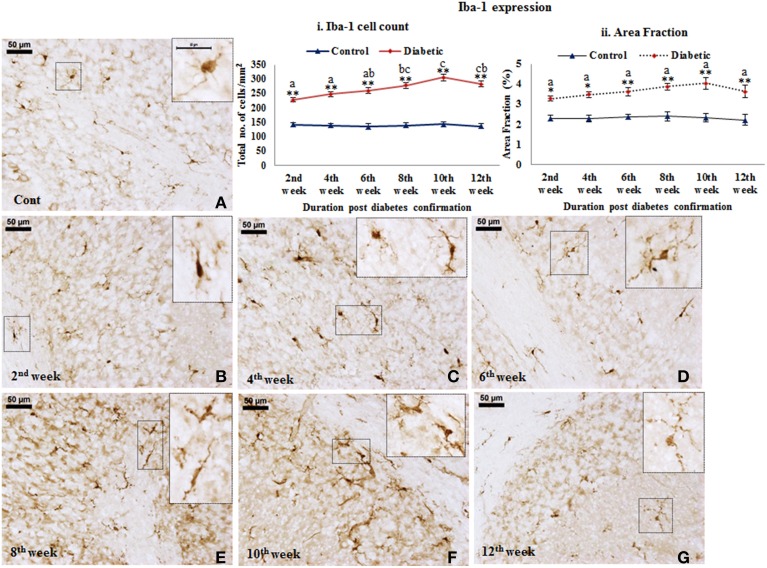
**Microglial activation followed with morphological transformation during diabetes**. Iba-1 immunohistochemical analysis of STZ-induced diabetic and control animals revealed the gradual microglial activation in the cerebellum of the diabetic animal upto 12 weeks **(B–G)** with having thick and reduced processes and irregular darkly stained cell body. While in controls **(A)** the microglia were in resting phase with thin and more number of processes and round cell body. Insets were showing the magnified images of Iba-1 expressing cells exhibiting the resting **(A)** and activated **(B–G)** morphology in cerebellum. Scale bar = 50 μm. Cell quantitation (i) and area fraction (ii) graphs were showing the diabetes associated elevation in the number of microglia and volumetric fraction from 2nd week onwards in comparison to controls. The increment in microglia population is indicative of proliferation following diabetes in cerebellum. Same alphabets on bars indicate non-significant differences between groups at the given time points (*p* ≤ 0.001). Value represents mean ± s.e.m. of the 30 readings/ animal/ time point. ^*^p ≤ 0.01, ^**^p ≤ 0.001 for comparison of diabetic group with the respective controls.

***Microglial activation.*** STZ-induced diabetes resulted in intense microglial activation and morphological transformation to activated phenotype in the cerebellum as shown by Iba-1 immunolabeling. In controls, microglia were seen with resting morphology having small, thin, multiple processes and round small cell body (Figure 5A). Following diabetes, an increase in Iba-1 expression was clearly evident in the cerebellar microglia. With the advancing diabetic duration, the resting microglia got transformed into activated microglia exhibiting thick and fewer numbers of processes with darkly stained Iba-1 positive irregular cell body (Figures 5B–G). Additionally, there was also a significant increase in microglial population in the diabetic rat cerebellum at all the time point studied, i.e., 2nd week [*F*_(11,359)_ = 9.533, *p* ≤ 0.001], 4th week [*F*_(11,359)_ = 11.995, *p* ≤ 0.001], 6th week [*F*_(11,359)_ = 13.822, *p* ≤ 0.001], 8th week [*F*_(11,359)_ = 15.490, *p* ≤ 0.001], 10th week [*F*_(11,359)_ = 17.953, *p* ≤ 0.001) and 12th week [*F*_(11,359)_ = 16.285, *p* ≤ 0.001] as compared to the respective controls indicating microgliosis in response to the alterations following diabetes (Figure 5i). Percentage of Iba-1 expression as area fraction is also increasing at 2nd week [*F*_(11,359)_ = 4.682, *p* = 0.044], 4th week [*F*_(11,359)_ = 5.817, *p* = 0.002], 6th week [*F*_(11,359)_ = 6.084, *p* = 0.001], 8th week [*F*_(11,359)_ = 7.145, *p* ≤ 0.001], 10th week [*F*_(11,359)_ = 8.352, *p* ≤0.001], and 12th week [*F*_(11,359)_ = 6.845, *p* ≤0.001] of diabetic cerebellum as compared to the controls (Figure 5ii).

#### Cell death following diabetes

**Figure 6:**
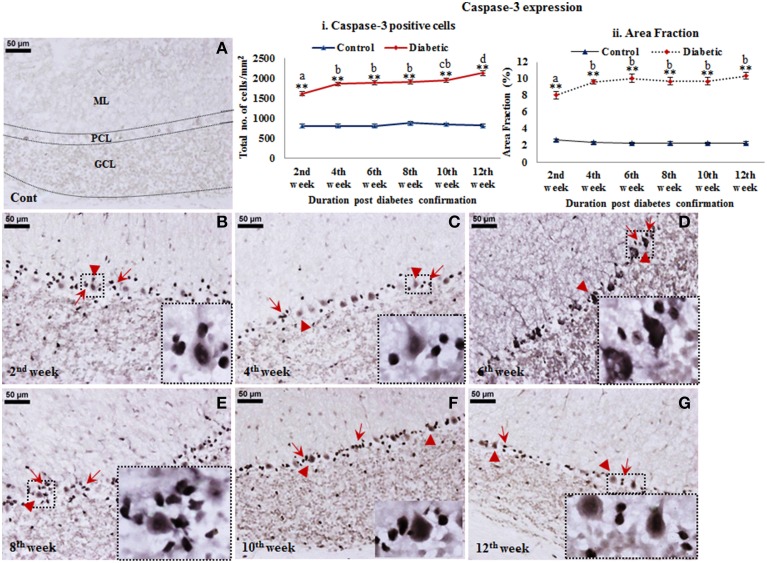
**Cell death detection in diabetic cerebellum**. Active caspase-3 Immunolabeling revealed the conspicuous cell death following diabetes in cerebellum. Apoptotic cells were clearly visible in all the three layers of the diabetic cerebellum at all the time points **(B–G)**. While in controls the labeling was negligible **(A)**. The effect of diabetes was clearly demarcated in the Purkinje cell layer as Bergmann glial cells (**B–G**; red arrows) surrounding Purkinje cells (**B–G**; red arrowheads) were also showing intense immunolabeling of active caspase-3 in comparison to the molecular and granule cell layer of cerebellum. Insets were showing the enlarged images of apoptotic Purkinje cell with surrounded Bergmann glial cells. Scale bar = 50 μm. Cell quantitation (i) and area fraction (ii) graphs were presenting the increased stature of active caspase-3 immunolabeled apoptotic cells in various layers of the cerebellum following diabetes. Quantitative assessment data were showing significant increment in apoptotic cell population and volumetric fraction at all the diabetic time points in comparison to controls. Same alphabets on bars indicate non-significant differences between groups at the given time points (*p* ≤ 0.001). Value represents mean ± s.e.m. of the 30 readings/animal/time point. ^**^p ≤ 0.001 for comparison of diabetic group with the respective controls.

A reflective cell death in cerebellum following STZ-induced diabetes upto 12 weeks was observed from active caspase-3 immunolabeling. Active caspase-3 positive cells were clearly evident in molecular layer, Purkinje cell layer and granule cell layer of the diabetic cerebellum in comparison to controls (Figure 6). Interestingly, active caspase-3 expression was more prominent in the nuclei of Purkinje cells and in surrounding Bergmann glial cell bodies in comparison to the molecular and granule cell layer. The inclination in Purkinje and Bergmann cell degeneration was constant at all the diabetic time points in comparison to controls. Quantitatively, caspase-3 positive cells were significantly increasing with advancing diabetic state, i.e., 2nd week [*F*_(11,359)_ = 17.409, *p* ≤ 0.001], 4th week [*F*_(11,359)_ = 22.529, *p* ≤ 0.001], 6th week [*F*_(11,359)_ = 23.258, *p* ≤ 0.001], 8th week [*F*_(11,359)_ = 22.187, *p* ≤ 0.001], 10th week [*F*_(11,359)_ = 23.848, *p* ≤ 0.001], and 12th week [*F*_(11,359)_ = 28.146, *p* ≤ 0.001] as compared to the respective controls (Figure 6i) suggesting a severe cellular degeneration in the cerebellar tissue. Percentage of area fraction of caspase-3 expression was also showing the similar trend. The increment of caspase-3 expression was consistent at all the diabetic time points, i.e., 2nd week [*F*_(11,359)_ = 16.397, *p* ≤ 0.001], 4th week [*F*_(11,359)_ = 22.076, *p* ≤ 0.001], 6th week [*F*_(11,359)_ = 23.570, *p* ≤ 0.001], 8th week [*F*_(11,359)_ = 22.544, *p* ≤ 0.001], 10th week [*F*_(11,359)_ = 22.569, *p* ≤ 0.001], and 12th week [*F*_(11,359)_ = 24.413, *p* ≤ 0.001] as compared to the controls (Figure 6ii).

#### Cellular degeneration following STZ-induced diabetes

**Figure 7:**
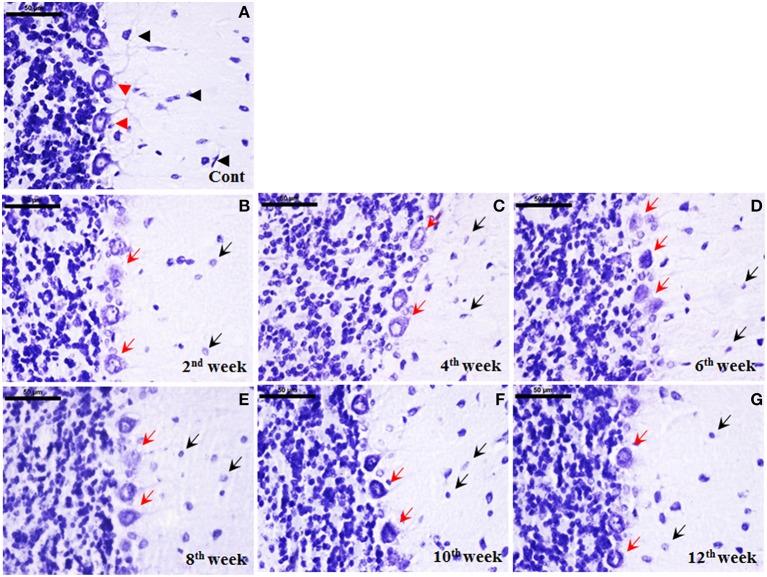
**Cellular degeneration in cerebellum following diabetes**. Histological cresyl violet (CV) staining depicted cellular degeneration in cerebellum in terms of lightly stained condensed nissl substance, loss of nucleus and reduced number of Purkinje cells (**B–G**; red arrows) following STZ-induced diabetes upto the 12 week. The degenerated purkinje cells were clearly demarcated in all the diabetic time points (red arrows). Additionally, cells in molecular and granule cell layer were reduced in number with small lightly stained cell bodies presenting chromatolysis following diabetes (**B–G**; black arrows) in comparison to the controls having darkly stained large sized cells (**A**; black arrowhead). In controls, Purkinje cells were darkly stained arranged in a uniform monolayer with centric nuclei (**A**; red arrowheads). Scale bar = 50 μm.

Histological staining with cresyl violet depicted a marked cellular degeneration in cerebellum following diabetes. Nissl bodies in control cerebellar cells were darkly stained with populous density. While following STZ-induced diabetes, a consistent loss of Nissl substance in cerebellar cells upto the 12th week was observed. Additionally, the effect of diabetes was more demarcated and regular in Purkinje cells in terms of both morphological alterations and cell number. In controls, darkly stained Purkinje cells were uniformly aligned presenting centralized nuclei (Figure 7A), while in diabetic cerebellum a marked Purkinje cell degeneration was observed with disorganized Purkinje cell layer devoid of nucleus and condensed lightly stained Nissl substance in cell body signifying abrupt accumulation of rough endoplasmic reticulum (RER; Figures 7B–G). With advancing diabetic state voids were also observed in the monolayer of Purkinje cells indicating the persistent Purkinje cell loss in cerebellum.

#### Histoarchitectural changes in the cerebellum during diabetes

**Figure 8:**
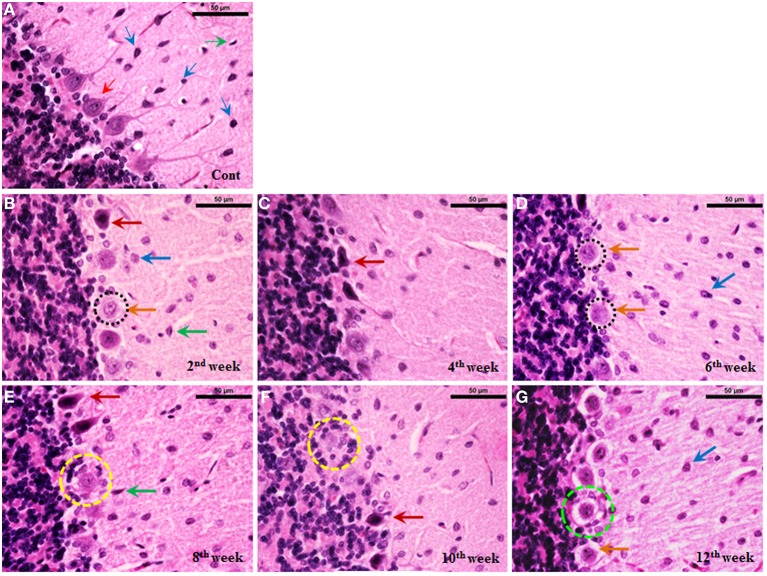
**Histoarchitectural alterations in cerebellum during diabetes**. Hematoxylin and eosin stained diabetic cerebellum presented a marked degeneration and cell death consistently upto the 12th week. Control cerebellar tissue presented intact cerebellar layers having normal Purkinje cells with dendrites and centrally placed nuclei (**A**; red thin arrow) and resting astroglia (**A**; blue thin arrow) and microglia (**A**; green thin arrow). Effect of diabetes was clearly evident in cerebellar cells presenting pathological signs of degeneration and cell death **(B–G)**. 2nd week onwards Purkinje cells were presenting chromatolysis, necrosis/apoptosis (**B–G**; red and orange thick arrows), pyknosis (**B–G**; red thick arrows), vacuolation (**G**; dotted green circle) and neuronophagia (**E,F**; dotted yellow circle). Astroglia (**B–G**; thick blue arrow) and microglia (**B–G**; thick green arrow) activation was also observed in cerebellar layers at all the diabetic time points. Scale bar = 50 μm.

Cerebellum of control rats stained with haematoxylin and eosin showed intact arrangement of molecular, Purkinje and granular cell layers with normal distribution of glial cells (Figure 8A). Purkinje cell monolayer was continuous with a centrally placed nucleus in the cell body. STZ-induced diabetic rats also showed intact arrangement of all the three layers. In contrast to control most of the Purkinje cells of the diabetic cerebellum were swollen, showing chromatolysis and vacuolation, suggesting necrotic cell death. This focal area of necrosis was surrounded by activated glial cells depicting the typical histomorphology of neuronophagia (Figures 8B–G). In addition, some Purkinje neurons presented shrunken and pyknotic appearance suggestive of apoptotic cell death (Figures 8B–G). Severity and incidence of dying or degenerating cerebellar cells increased with the advancing stage of diabetes. Activation of astroglia and microglia was evident from 2nd week and remained consistent till 12 week of the diabetic rat cerebellum. In the later stages activated, glial cells were circumscribing the necrotic/apoptotic neurons (Figures 8C–G). Interestingly, a discontinuity in the Purkinje cells monolayer indicates a correlation with the severity of degenerative histoarchitectural alterations following diabetes. Details of histopathological findings are presented, in the summary incidence table (Table 1).

**Table 1:** **Summary incidence table of lesions in control and STZ-induced diabetic rat cerebellum**.

	**Control**	**2nd week**	**4th week**	**6th week**	**8th week**	**10th week**	**12th week**
Ballooning of purkinje cell	−	++	++	++	++	++	++
Chromatolysis	−	++	++	+++	++++	++++	++++
Vacuolation	−	−	++	++	+++	+++	++++
Necrosis/Apoptosis	−	++	++	+++	+++	++++	++++
Pyknosis/Dark purkinje cell	−	++	++	+++	+++	++++	++++
Gliosis	−	+	++	++	+++	++++	++++
Neuronophagia	−	−	++	++	+++	++++	++++

−, Nil (less than 5%); +, minimal (5–10%); ++, mild (10–20%); +++, moderate (20–30%); ++++, severe (more than 30%).

#### Effect of diabetes on astroglial glutamate transporter

**Figure 9:**
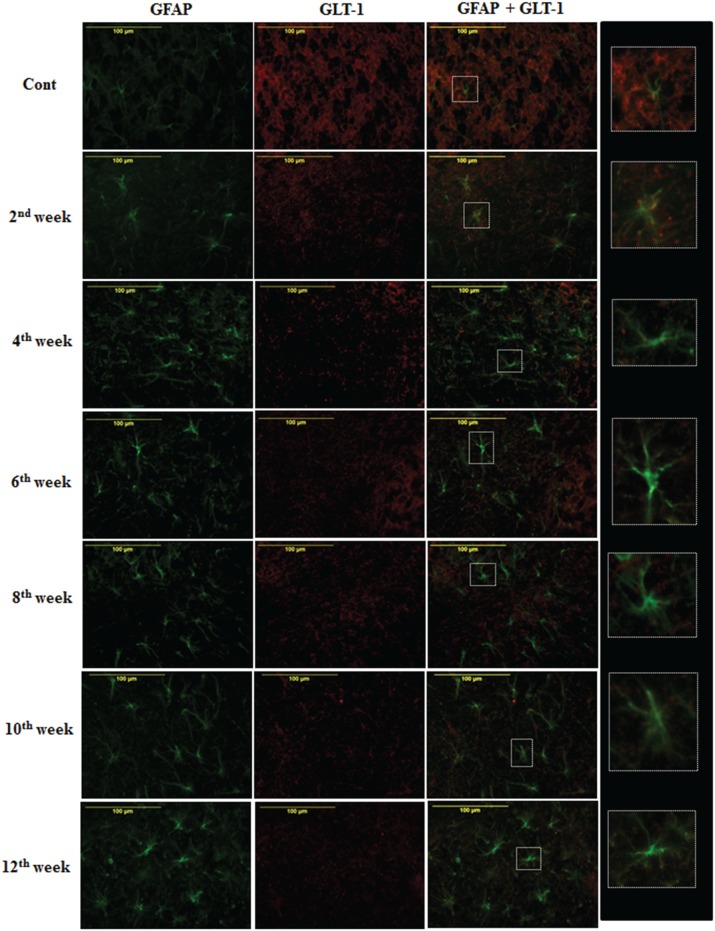
**Diabetes caused reduction in astroglial glutamate transporter GLT-1**. Co-immunolabeling of GFAP + GLT-1 showing a marked reduction in astroglial glutamate transporters following diabetes in cerebellum. The GLT-1 was clearly localized with the processes of GFAP expressing astroglia in controls tissue of cerebellum. Insets were showing the enlarged co-labeled (GLT-1 and GFAP) image of astroglia (A). Scale bar = 100 μm.

**Figure 10:**
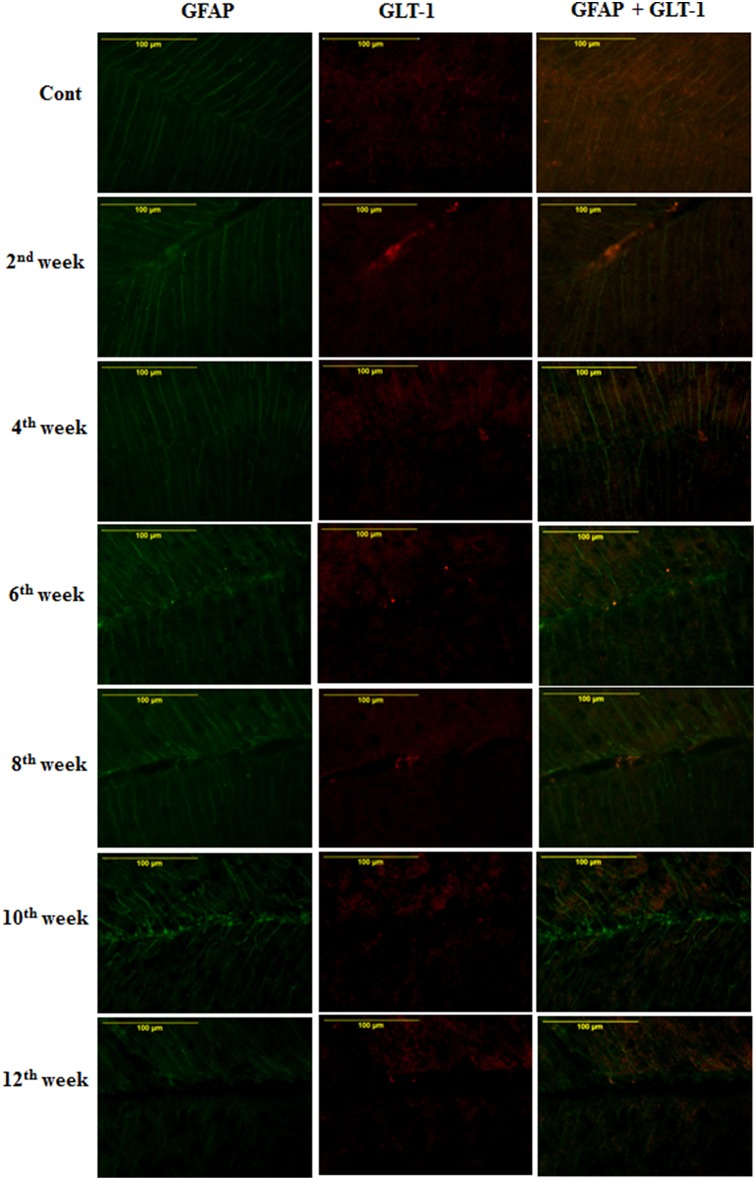
**Effect of diabetes on the glutamate transporters of the BGL**. Following STZ-induced diabetes the GLT-1 expression was reduced in Bergmann glial fibers in comparison to controls consistently in all the diabetic time points. Scale bar = 100 μm.

**Figure 11:**
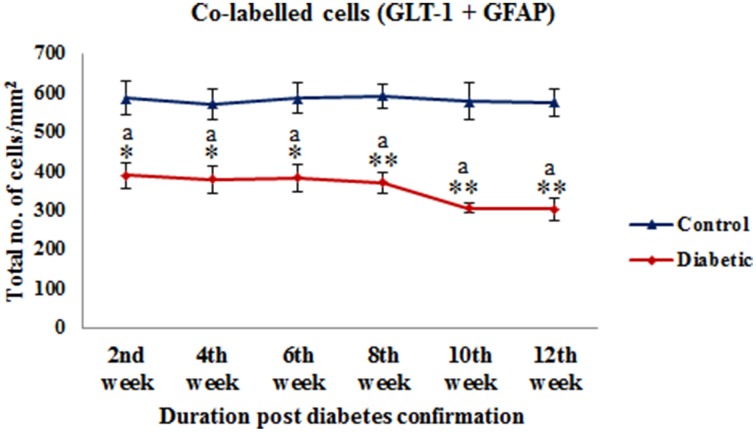
**Quantification of co-labeled cells (GLT-1 + GFAP) in various layers of the cerebellum following diabetes**. There is significant reduction in the population of co-labeled cells at all the diabetic time points as compared to their respective controls. Same alphabets on bars indicate non-significant differences between groups at the given time points (*p* ≤ 0.001). Value represents mean ± s.e.m. of the 30 readings/ animal/ time point.^*^p ≤ 0.01, ^**^p ≤ 0.001 for comparison of diabetic group with the respective controls.

On the assessment of GLT-1 transporters presence on astroglia and BGF, we recorded a reduced co-immunolabeling of glutamate transporters on both astroglia (Figure 9) and BGF (Figure 10) following diabetes in cerebellum in all the diabetic time points. While in controls, GLT-1 marker was noticeably co-labeled with GFAP expressing astroglia and BGF. Quantitation of GLT-1 + GFAP co-labeled cells also showed a significant reduction in the GLT-1 co-labeling with GFAP immunoexpression upto the 12th week of diabetes confirmation, i.e., 2nd week [*F*_(11,359)_ = 5.705, *p* = 0.003], 4th week [*F*_(11,359)_ = 5.560, *p* = 0.005], 6th week [*F*_(11,359)_ = 5.850, *p* = 0.002], 8th week [*F*_(11,359)_ = 6.452, *p* ≤ 0.001], 10th week [*F*_(11,359)_ = 7.883, *p* ≤ 0.001], and 12th week [*F*_(11,359)_ = 7.904, *p* ≤ 0.001] as compared to their respective controls (Figure 11). Thus, reduced expression of GLT-1 co-labeled GFAP^+^ Bergmann glial fibers and astroglial cells (white matter and granule cell layer) in the diabetic cerebellum indicates the altered glutamate transportation in the cerebellum as a detrimental effect of increased blood-glucose level.

## Discussion

Diabetes involves various comorbid complications such as learning and memory impairment (Biessels et al., 1996; Popoviç et al., 2001), reduced muscular strength (Andersen et al., 2005), behavioral deficits (Sweetnam et al., 2012) and more. The causative factors for these alterations range from structural, cellular to neurochemical changes leading to direct neuronal damage and thus loss of information processing (Thorré et al., 1997; Alvarez et al., 2009). STZ-induced animal model is widely used to elucidate the diabetes associated complications. The studies related to cognition and behavior following diabetes is limited to the hippocampal atrophy and allied cellular changes. A sole study reported the role of cerebellar alterations in behavioral deficits following hypoglycaemia in terms of decreased GABA receptor and CREB expression (Sherin et al., 2010). Cerebellum is associated with emotion, cognition and behavior (Rapoport et al., 2000; Schmahmann and Caplan, 2006) and alterations to the cerebellum lead to motor deficits, dementia, schizophrenia and other psychiatric disorders (Baldaçara et al., 2008; Sui and Zhang, 2012). In the present study, we have demonstrated the influence of glial activation, cell death, and loss of glutamate transporters in the diabetic rat cerebellum on reduced motor and muscle activity.

The STZ-induced rats presented the characteristic signs of diabetes as regular body weight loss and subsequent high blood-glucose level due to the loss of pancreatic beta cells as has been reported earlier (Junod et al., 1969). During diabetes, the glucose utilization in the brain gets decreased (McCall, 1992), which makes the brain more vulnerable to the critical pathological events. A significant cellular degeneration and/or cell loss was observed in the cerebellum of STZ-induced diabetic animals in both immunohistochemical and histological study consistently upto the 12th week of diabetic duration. Active caspase-3 positive cells and were successively increasing in all the layers of the cerebellum. The cellular degeneration of both Purkinje and Bergmann glial cells in the cerebellum indicates the compromised motor information processing following diabetes as they both together constitute the unit of synaptic information transmission (Bellamy, 2006). The deleterious effect of diabetes and hyperglycemia as cell death was previously well reported with underlying mechanisms of activation of p53 transcription factor, intrinsic cell death pathway and decreased IGF-I level due to increased blood glucose (Yamaguchi et al., 2001; Muranyi et al., 2003; Lechuga-Sancho et al., 2006). Studies suggested that p53 transcription factor is involved in the activation of caspase-3, 9, and 6 (Culmsee and Mattson, 2005) and also takes part in some forms of cerebellar cell death (Inamura et al., 2000). Similarly increased blood-glucose level as observed in this study is expected to reduce the IGF-I level in cerebellar granule neurons (Linseman et al., 2002). Constitutively, the reduced IGF-I level due to high blood-glucose level or activation of p53 transcription factors or intrinsic cell death pathway might be the potential cause behind the sequential apoptotic cell death following diabetes in the cerebellum as reported in this study.

Glial cells are susceptible to any change or disruption in the brain. In response to adverse state, glial cells transform their morphology and proliferate to combat the baleful condition (Patro et al., 2005; Verkhratsky et al., 2012). In the current study, following diabetic condition astroglia and microglia were also showing alterations in terms of their activated morphology and increased population upto the 12th week of diabetes in the cerebellum. This activation might be a customized approach to modulate the effect of excessive cell death following diabetes. However, our present astroglial observations in the diabetic cerebellum are differed from the previous studies revealing the decrement in astrocyte GFAP levels in the cerebellum both in terms of cell number and labeling intensity (Coleman et al., 2004; Guven et al., 2009) at 4/8th weeks of diabetic duration. As astroglia play, a compelling role in transporting glucose and its metabolites to neurons, thus alterations in glucose level following diabetes might be a possible reason of astroglial dystrophy in the cerebellum.

Microglial activation and proliferation following diabetes in the cerebellum was previously well reported following diabetic retinopathy (Zeng et al., 2000), acute cerebral infarction in presence of diabetes mellitus (Li et al., 2011) and in diabetic hyperalgesic rat spinal cord (Daulhac et al., 2006). Similar microglial activation and increment in population was also observed in the present study of cerebellum following STZ-induced diabetes. Our previous studies showed that being the prime immune cell in brain, microglia gets readily activated in response to any injury (Saxena et al., 2007; Patro et al., 2010b), inflammation (Patro et al., 2010a, 2013) and metabolic disorder (Nagayach et al., 2014). During immune breaching or cell death, microglia becomes activated to protect and repair the damaged tissue via removing the dying cell debris and facilitating the healing process (Hanisch and Kettenmann, 2007; Kettenmann et al., 2011). On the contrary, microglial activation is also responsible in aggravating the neurodegeneration (Block and Hong, 2005; Venero et al., 2011). Therefore, microglia activation following diabetes in the cerebellum as shown in this study might be in response of cell death for providing the potential damage control or possibly microglial activation was itself inducing the cell death via generating various immune mediators.

Astroglia actively participates in the synaptic transmission related to the modulation of synaptic information processing via astroglial glutamate transporters forming tripartite synapse (Araque et al., 1999; Santello et al., 2012). On neuron-glia, synaptic junction glutamate transporters help in regulating the level of extracellular glutamate and minimize the glutamate excitoxicity. Likewise in cerebellum, Bergmann glial cells possess glutamate transporters (GLAST/GLT-1), that not only help in glutamate uptake (Rothstein et al., 1994; Bellamy, 2006) but also remove the excess of glutamate from the synaptic cleft and act as signal transducer (López-Bayghen et al., 2007). In cerebellum unlike GLAST, GLT-1 is relatively low in density but it plays a similar and imperative role in uptaking of glutamate (Takayasu et al., 2009). In this study we observed that with advancing diabetic state glutamate transporters, i.e., GLT-1 were reducing on astroglia and Bergmann glial fibers. Reduction in GLT-1 mRNA level is well reported in various brain disorders like ischemia, amyotrophic lateral sclerosis, Alzheimer's disease and Huntington's disease (Torp et al., 1995; Sims and Robinson, 1999; Gegelashvili et al., 2001) with subsequent consequences of secondary neuronal cell death, excessive activation of glutamate receptors, abnormal neuronal activity and ensuing excitotoxic degeneration (Rothstein et al., 1996; Tanaka et al., 1997). As the present results markedly showed the hypertrophied activated astroglia and fragmented processes of Bergmann glial fiber, which is possibly due to the degeneration of Bergmann glial cell bodies, and the glutamate transporters are localized on the processes of astroglia and BGF, so it might be possible that fragmentation of astroglia and BGF processes were causing the loss of glutamate transporters and commencing the glutamate excitotoxicity in the cerebellum. Another possibility for selective loss of GLT-1 on astrocytes was due to the diabetes associated oxidative stress as it causes the oxidation of glutamate transporters (Trotti et al., 1998).

Purkinje cell as the prime relay neurons of the cerebellum plays an imperious role in motor coordination and learning. As reported earlier the inevitable loss of Purkinje cell resulted in various motor disorders including autism, ataxia and Huntington's disease (Whitney et al., 2008; Matilla-Dueñas et al., 2012; Rüb et al., 2013). Consistent with our immunohistochemical cell death study, histological data also depicted the specific Purkinje cell degeneration and cell loss in cerebellum extending the aura of cellular alterations following diabetes. The loss of Purkinje cell might be explained as an effect of altered cerebellar microenvironment due to increased blood-glucose level and glial activation. Possibly, the degeneration of cerebellar cells and allied alterations in communication elements (Purkinje neuron, glia, glutamate transporters, etc.) are instigating the alterations in the motor information processing in the cerebellum.

Alterations in motor behavior following diabetes was previously well reported both in human and animal models with underlying causes of increased blood glucose level associated micro- or macrovascular complications (Demirbüken et al., 2012) and altered neurotransmission (Sherin et al., 2012). In our study, the diabetic animals were showing reduced falling latency on Rotarod and poor or weak muscular strength in grip strength meter indicating impaired motor/muscle activity upto the 12 weeks of diabetic duration. Cerebellum participates in the control and coordination of motor/muscle activity, which is possibly controlled and maintained by the neuron-glia shuttle. In the cerebellum, the main interceptive unit of motor control, i.e., Purkinje neurons are surrounded by the Bergmann cell bodies, and its dendritic synapses are enwrapped by the Bergmann glial fibers expressing GLT-1 on its surface membrane (Rothstein et al., 1994; Chaudhry et al., 1995). Thereby, it was suggested that excitatory synapses of Purkinje neurons and associated cerebellar motor control are regulated by the glia-neuron circuitry via glutamate transporters present in BGF (Bellamy and Ogden, 2005). A study also suggested that the Bergmann glia as an astrocyte in molecular and Purkinje layer of the cerebellum become activated in response of motor behavior (Nimmerjahn et al., 2009). Therefore, severe Purkinje and Bergmann glial cell loss, fragmentation of astroglia and BGF processes with reduced expression of glutamate transporter on astroglia and Bergmann glia might be affecting the interception between the cerebellar cells resulting in motor deficits following diabetes. To the best of our knowledge, this is the first study elucidating the influence of glial activation, cellular degeneration and glutamate toxicity on altered behavioral activity following STZ-induced diabetes in rats.

**Figure 12:**
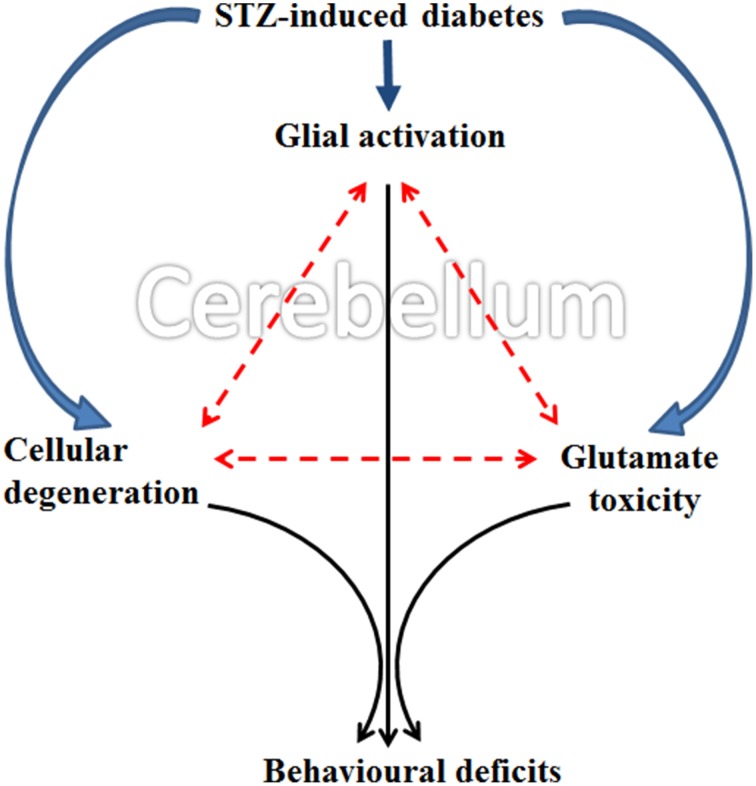
**Schematic representation showing possible occurrence of events in cerebellum following STZ-induced diabetes**. Diabetes simultaneously induce glial activation, cellular degeneration and glutamate toxicity in cerebellum and the subsequent triangular associative interplay between these three factors resulted in motor deficits following diabetes.

The present collective evidence grounds the consideration that diabetes induces (a) cellular degeneration, (b) glial activation and (c) and reduced glutamate transportation in the cerebellum. Later, the triangular associative interplay between these three factors in cerebellum results in motor deficits following diabetes (Figure 12). Although Western blot would have been an added advantage, with the data exclusively derived from the histology, immunohistochemistry and cellular quantifications, we conclude that the possible acute involvement of glial cells and associated neurochemical changes in cerebellum following diabetes offers an upcoming therapeutic approach for developing the treatment of diabetes associated motor behavior deficits.

### Conflict of interest statement

The authors declare that the research was conducted in the absence of any commercial or financial relationships that could be construed as a potential conflict of interest.
